# High Sensitive Sensor Fabricated by Reduced Graphene Oxide/Polyvinyl Butyral Nanofibers for Detecting Cu (II) in Water

**DOI:** 10.1155/2015/723276

**Published:** 2015-01-28

**Authors:** Rui Ding, Zhimin Luo, Xiuling Ma, Xiaoping Fan, Liqun Xue, Xiuzhu Lin, Sheng Chen

**Affiliations:** ^1^College of Environmental Science and Engineering, Fujian Normal University, 8 Shangsan Road, Fuzhou 350007, China; ^2^Jiangsu Key Laboratory for Organic Electronics & Information Displays and Institute of Advanced Materials (IAM), Nanjing University of Posts and Telecommunications, Nanjing 210046, China; ^3^College of Chemistry and Chemical Engineering, Fujian Normal University, 8 Shangsan Road, Fuzhou 350007, China; ^4^Department of Biology and Chemistry Engineering, Fuqing Branch of Fujian Normal University, 1 Longjiang Road, Fuqing 350300, China

## Abstract

Graphene oxide (GO)/polyvinyl butyral (PVB) nanofibers were prepared by a simple electrospinning technique with PVB as matrix and GO as a functional nanomaterial. GO/PVB nanofibers on glassy carbon electrode (GCE) were reduced through electrochemical method to form reduced graphene oxide (RGO)/PVB nanofibers. The morphology and structure of GO/PVB nanofiber were studied by scanning election microscopy (SEM), transmission electron microscopy (TEM), and Fourier transform infrared (FTIR). RGO/PVB modified GCE was used for fabricating an electrochemical sensor for detecting Cu (II) in water. The analysis results showed that RGO/PVB modified GCE had good analytical results with the linear range of 0.06–2.2 *μ*M, detection limit of 4.10 nM (*S*/*N* = 3), and the sensitivity of 103.51 *μ*A·*μ*M^−1^·cm^−2^.

## 1. Introduction

With the development of the industrialization, more and more containments caused the serious environmental problems. Among these pollutants, heavy metals have attracted great attention for they are harmful to human beings due to their toxicity, cumulative and nobiodegradability. Copper is one of the essential elements for human beings, but excessive intake of this element can result in certain diseases like Wilson's disease and Menke's syndrome, and so forth [1]. However, the casual wastewater discharge from mining, machinery manufacturing, and metal smelting led to a serious copper pollution in water. Therefore, search for real-time, rapid, and sensitive detection of Cu (II) in water system is of significance. A variety of methods have been established to detect Cu (II), such as flame atomic absorption spectrometry [2], UV-vis spectrophotometry [3], and inductively coupled plasma mass spectrometry [4]. These traditional detection methods have achieved rather good detection limit. However, they need a tedious pretreatment or result in spectroscopic interference. On the contrary, the electrochemical methods [5–10] have been widely applied for detecting trace ions in water because they are sensitive, accurate, inexpensive, rapid, and portable. For example, a functionalized carbon nanotubes paste electrode (CNPE) modified with crosslinked chitosan was used for detecting Cu (II) with the limit detection of 10 nM and the linear range of 0.079–16 *μ*M [11]. An electrochemical sensor based on the functionalized polypyrrole (PPy) nanotube modified with tripeptide (Gly-Gly-His) was developed for detecting Cu (II) with the detection limit of 46 nM and the linear range of 0.1–30 *μ*M [12]. In order to improve the sensitivity and the analytical performance, new nanomaterials will be needed to construct the sensor for Cu (II) detection.

Graphene, a single layer of sp^2^ bonded carbon atoms arranged into a honeycomb structure with extraordinary electronic transport properties, excellent electrocatalytic activities [13], high modulus, and high specific surface area [14, 15], has stimulated extensive attention since its discovery [16]. It has been applied in many technological fields, including photodetectors [17, 18], antibacterial materials [19, 20], fuel cells [21, 22], lithium ion batteries [23], and supercapacitors [24, 25], and has been developed as an advanced nanoelectrocatalyst for constructing electrochemical sensors [26, 27]. The used graphene in electrochemistry was usually produced from the reduction of graphene oxide. However, reduced graphene oxides (RGO) tend to agglomerate during the reduction of graphene oxide (GO) because of the van der Waals and *π*-*π* stacking interactions among individual graphene sheet interactions, which may reduce the conductivity and its specific surface area, and thus result in weakening the performance of sensor based on RGO [28, 29]. Electrospinning is a simple and cost-effective technique to fabricate one-dimensional nanostructures with high surface area and porosity [30], such as the electrospinning carbon nanofiber, which has been a promising candidate for designing gas sensors [31], chemical sensors [32–34], and biosensors [35] due to its remarkable electronic properties, electrochemical performances, and mechanical strength [36]. Through electrospinning technique, the made RGO can avoid agglomerating.

In this study, a novel Cu (II) sensor was fabricated based on RGO/PVB nanofibers, which was prepared by electrospinning the composite of GO and PVB and in situ electrochemical reduction of GO/PVB nanofibers. The RGO/PVB nanofibers modified glassy carbon electrode (GCE) was used for Cu (II) detection by differential pulse anodic stripping voltammetry (DPASV). The as-fabricated sensor based on RGO/PVB nanofibers showed good analytical performance with the linear range of 0.06–2.2 *μ*M, a low detection limit of 4.1 nM (*S*/*N* = 3), high sensitivity of 103.51 *μ*A·*μ*M^−1^·cm^−2^, good selectivity, and excellent reproducibility (RSD = 0.49%).

## 2. Materials and Methods

### 2.1. Chemical Reagents

Sodium acetate and glacial acetic acid (99.5%) were purchased from Guoyao Chemicals Co. Ltd. Nafion (5%) was a product of Sigma-Aldrich. PVB (butyral content: 66%–75%), potassium hydroxide, and N,N-dimethylformamide were obtained from Guoyao Chemicals Co. Ltd. All the other reagents are of analytical grade. The used water was deionized water.

### 2.2. Preparation of GO/PVB Nanofiber

The uniform electrospinning solutions were prepared by mixing GO, PVB, and N,N-dimethylformamide (DMF). DMF was used as a solvent. The solution was placed in a 5 mL syringe with a metallic needle of 0.4 mm of inner diameter. The syringe was fixed horizontally and connected with a high voltage power supply (Tianjin Technical Corp.), which could generate DC voltage up to 30 KV. The supplied voltages between the tip and collector were set at 16 KV with a tip-to-collector distance of 15 cm. After several minutes, PVB/GO nanofiber-modified GCE (GCE/PGNF) was prepared and dried at 60°C for next use.

### 2.3. Fabrication of RGO/PVB Modified GCE

GO/PVB nanofiber was fixed on GCE through dropping 3 *μ*L 0.5% Nafion on the surface of GCE/PGNF. Then GCE/PGNF was immersed into the 0.1 M KOH solution and reduced by scanning cyclic voltammetry with the Pt wire as counter electrode and Ag/AgCl (saturated by KCl) as reference electrode. The formed GCE modified with RGO/PVB nanofiber (GCE/PRGNF) was washed with deionized water and stored for Cu (II) detection.

### 2.4. Detection of Cu (II) with GCE/PRGNF

**Figure 1:**
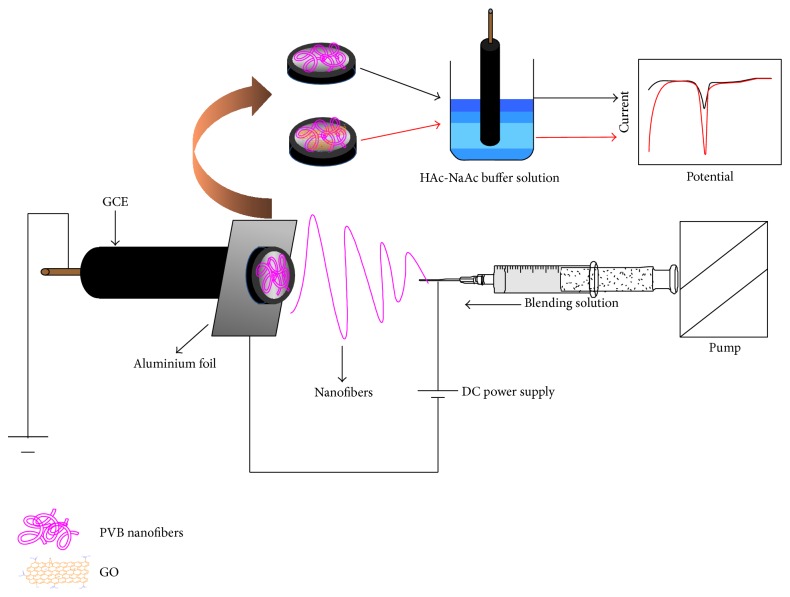
Schematic illustration of electrospinning device.

GCE/PRGNF was immersed into HAc-NaAc buffer solution (pH 4.4) with various concentrations of copper ions for several minutes, the electrochemical response was measured by DPASV with a scanning potential range from −0.6 to 0.6 V, a step voltage of 4 mV, an impulse amplitude of 50 mV, a pulse width of 0.06 s, and a pulse separation of 0.20 s, and the GCE/PRGNF was accumulated for 270 s with stirring under the constant potential of −0.5 V. The setup of the process used in the study is shown in Figure 1. After each DPASV measurement, the electrode was rinsed by i-t method at 0.60 V, placed in blank solution for 30 s for removing the sediment that adsorbed on the surface of the electrode and restoring the catalytic activity of the electrode. All the experiments were conducted at room temperature without deoxygenization. Nanofibers without GO or RGO were used for control experiments under the same condition.

## 3. Results and Discussion

### 3.1. Characterization of GO/PVB Nanofibers

**Figure 2:**
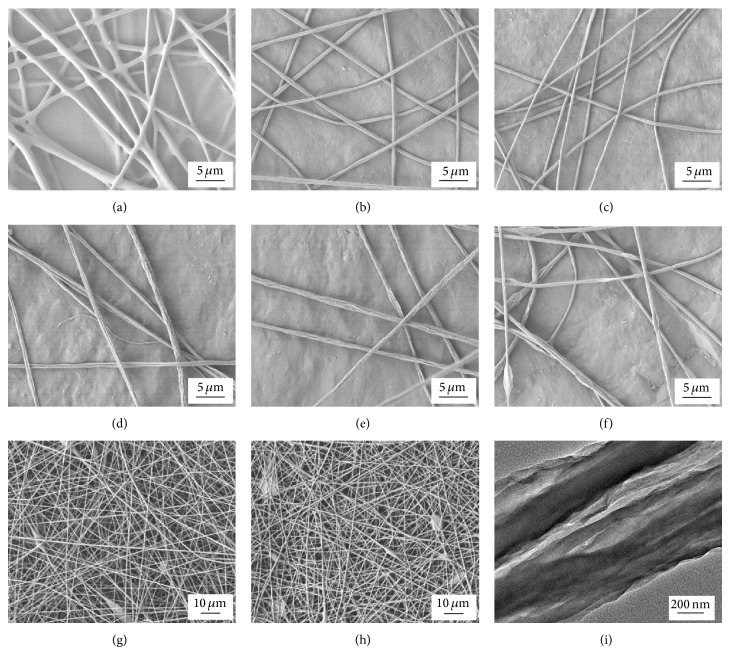
((a)–(f)) SEM images of electrospun GO/PVB nanofibers ((a) 0.8125%; (b) 1.625%; (c) 3.25%; (d) 4.875%; (e) 5.25%; (f) 6.875% GO); SEM images of electrospun GO/PVB nanofibers (g) before and (h) after the oscillation in water for 5 h; (i) TEM image of GO/PVB fiber.

The morphologies of the GO/PVB nanofibers were observed by scanning electron microscopy (SEM). GO/PVB nanofibers prepared with different concentrations of GO (0.8125%, 1.625%, 3.25%, 4.875%, 5.25%, and 6.875%) were explored. As shown in Figure 2, PVB/GO nanofiber (0.8125% GO) (Figure 2(a)) showed more uniform morphology of fibers. With the increase of the mass ratio of GO, the nanofibers became rather rough (Figure 2(b)), or even reunited (Figure 2(f)), because GO was over the solubility limit of DMF. Compared with Figure 2(g), the nanofibers did not change (Figure 2(h)) after stirring in water as PVB was nonhydrophilic. The representative TEM images of the GO/PVB nanofibers showed that GO was relatively dispersed in the nanofibers without any agglomeration.

**Figure 3:**
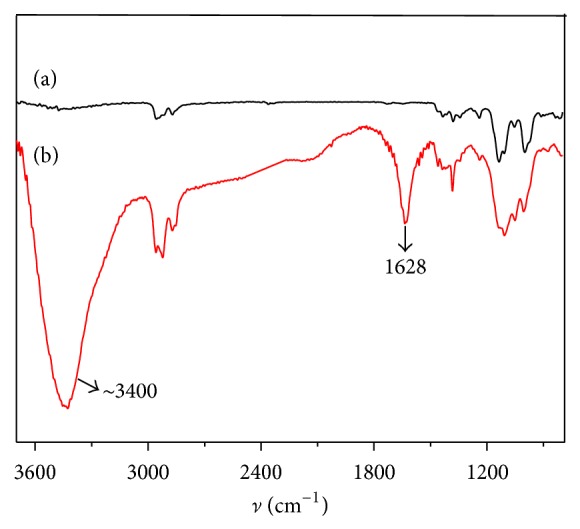
FTIR spectra of PVB (a) and GO/PVB (b) nanofibers.

Figure 3 showed the FTIR spectra of PVB nanofibers (Figure 3(a)) and PVB/GO nanofibers (Figure 3(b)). Compared with the spectrum of PVB nanofibers, the spectrum of PVB/GO nanofibers showed the intense absorption at 1628 cm^−1^ and 3400 cm^−1^. 1628 cm^−1^ can be assigned to the stretching vibration of epoxy group and carbon frame of GO. 3400 cm^−1^ corresponds to stretching vibration of hydroxy of GO. FTIR characterization confirms the dispersion of GO on the PVB matrix.

### 3.2. Electrochemical Detection of Cu (II) with GCE/PRGNF

**Figure 4:**
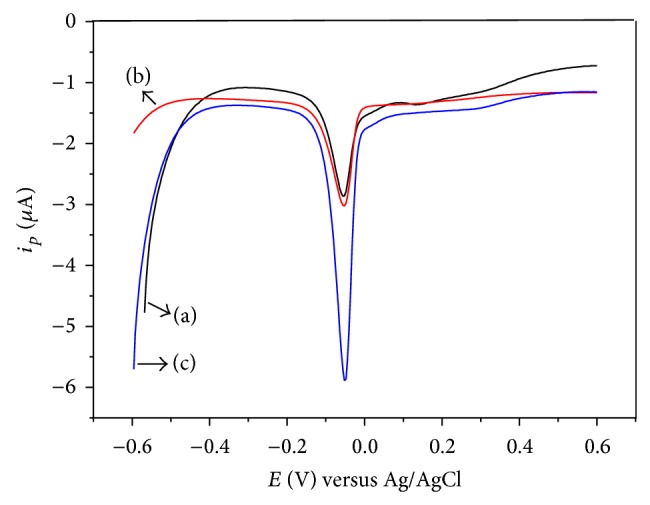
Differential pulse anodic stripping voltammetry of Cu (II) on GCE (a), GCE/PVB (b), and GCE/PRGNF (c).

Bare GCE, GCE/PVB, and GCE/PRGNF were used for detecting Cu (II) by DPASV. The measurements were carried out using a solution with 6.0 × 10^−7^ mol·L^−1^ of Cu (II) in 0.1 mol·L^−1^ of NaAc-HAc (pH 4.4), the accumulation potential range from −0.6 to 0.6 V, and accumulation time of 270 s. As shown in Figure 4, the anodic peak current response of the GCE/PRGNF (Figure 4(c)) was 5.9 *μ*A, which is higher than bare GCE (Figure 4(a)) (2.8 *μ*A) and GCE/PVB (Figure 4(b)) (3.0 *μ*A), indicating that RGO/PVB nanofiber can improve electroanalytical responses for Cu (II). It can be explained by two beneficial factors. One is the high conductivity of RGO in RGO/PVB nanofibers, which promotes the electron transfer in the procedure of detection. The other is the active sites of RGO in RGO/PVB nanofibers, which can absorb Cu (II) for increasing the amount of electrochemical reaction and enhance the current response for detecting Cu (II).

### 3.3. Optimization of Detection Conditions

**Figure 5:**
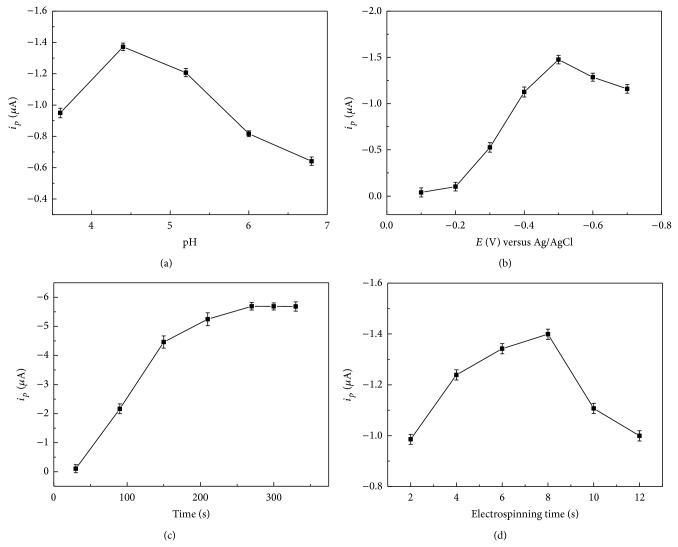
The effect of pH (a), accumulation potential (b), accumulation time (c), and electrospinning time (d) on the stripping peak currents of 6.0 × 10^−7^ mol·L^−1^ Cu (II).

The influences of detection conditions such as pH, accumulation potential, accumulation time, and electrospinning time were investigated. As can be seen in Figures 5(a) and 5(b), GCE/PRGNF had the best performance when the pH was 4.4 and the potential was −0.5 V. Therefore, 0.1 mol·L^−1^ of NaAc-HAc (pH 4.4) was selected for the best supporting electrolytes and −0.5 V was chosen for next experiments.

Figure 5(c) displayed the influence of accumulation time for stripping peak currents. With the increase of accumulation time, the stripping peak currents increased and reached to the maximum value after 270 s.

The electrospinning time was studied in the range of 2–12 s. As shown in Figure 5(d), when the electrospinning time was 8 s, the response current was highest since there was not enough PVB/RGO nanofiber on the surface of GCE when the electrospinning time was too short. If electrospinning time was too long, there was too much PVB/RGO, which influenced the transfer of electron on the surface of the modified GCE.

### 3.4. Reproducibility of GCE/PRGNF

The repeated use of GCE/PRGNF was examined by the i-t measurement of detecting Cu (II).  Table 1 showed the change of peak currents after 10 determinations of 6.0 × 10^−7^ mol·L^−1^ Cu (II) using the same GCE/PRGNF. The relative standard deviation was 0.49%, indicating that the GCE/PRGNF has an excellent repeated performance for sensing Cu (II).

### 3.5. Anti-Interference of GCE/PRGNF

In order to detect the identification performance of GCE/PRGNF, 6.0 × 10^−7^ mol·L^−1^ Cu (II) solutions coupled with different metal ions commonly present in natural water were used to research the anti-interference of GCE/PRGNF. The results were shown in Table 2. There was less than ±5% of interference for GCE/PRGNF towards Na (I), K (I), Ca (II), Mg (II), Mn (II), Cd (II), and Zn (II), except Pb (II). The reason for the influence of Pb (II) is that the mixed layer formed on the surface of electrode in the process of the enrichment of Pb (II) and Cu (II), which changes the performance of the electrode. The interference of Pb (II) was about 5.1%. Therefore, GCE/PRGNF was suitable for the determination of Cu (II) in real water samples with some pretreatments.

### 3.6. Detection of Cu (II) with GCE/PRGNF

**Figure 6:**
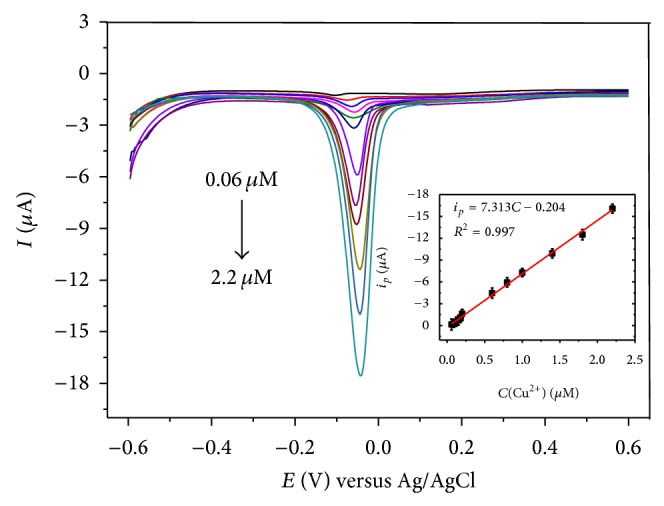
Differential pulse stripping voltammetry of GCE/PRGNF in 0.1 M NaAc-HAc (pH 4.4) with various concentrations of Cu (II) (from 0.06~2.2 *μ*M). Inset is the calibration curve of the stripping peak currents versus the concentrations of Cu (II).

Figure 6 showed the stripping voltammograms under optimized conditions with the concentration of Cu (II) from 0.06 to 2.2 *μ*M and the corresponding calibration curve of the stripping peak current versus the concentrations of Cu (II) (inset). The GCE/PRGNF showed good linear detection range from 0.06 to 2.2 *μ*M with a detection limit of 4.1 nM (*S*/*N* = 3) and a sensitivity of 103.51 *μ*A·*μ*M^−1^·cm^−2^. Compared with other nanomaterials modified electrodes [11, 12], PVB/RGO nanofibers modified GCE shows a low detection limit and a higher sensitivity.

## 4. Conclusions

Electrospinning technique was used to construct the hybrid nanofiber of GO and PVB. PVB/RGO nanofiber modified GCE was fabricated through electrochemical reduction of PVB/GO nanofiber modified GCE and applied for detection of Cu (II) in water. The PVB/RGO nanofibers modified GCE displays good analytical performance including wide linear range, low detection limit, high sensitivity, good repeatability, and anti-interference ability towards other metal ions. The composite of RGO into PVB nanofibers avoids the agglomerate of RGO and overcomes the defect of water-soluble polymer electrospinning membrane unfitted for application under high-humidity environment. The fabricated sensor based on PVB/RGO nanofibers is promising in the determination of trace Cu (II) in real samples.

## Figures and Tables

**Table 1:** *i*_*p*_ of the GCE/PRGNF responding to the 6.0 × 10^−7^ mol·L^−1^ Cu (II).

*n*	1	2	3	4	5	6	7	8	9	10
*i*_*p*_	0.461	0.466	0.460	0.463	0.463	0.460	0.466	0.462	0.464	0.461
(*μ*A)										

**Table 2:** Anti-interference of GCE/PRGNF.

Concentration (mol·L^−1^)	Species	Interference (%)
3.0 × 10^−4^	Na^+^	−1.6
	K^+^	−0.23
	Ca^2+^	−2.7
	Mg^2+^	+0.2

6.0 × 10^−5^	Mn^2+^	−3.1
	Cd^2+^	−2.4

1.2 × 10^−5^	Pb^2+^	−5.1
	Zn^2+^	−3.2
